# *Aspergillus foetidus* as a potent producer for β-galactosidase utilizing lemon peels and coffee waste powder: production optimization, purification, kinetic and thermodynamic characterization

**DOI:** 10.1186/s12934-024-02600-0

**Published:** 2024-12-17

**Authors:** Walaa A. Abdel Wahab, Shireen A. A. Saleh, Nermeen H. Elzairy, Samia A. Ahmed, Eman R. Zaki, Walaa H. Salama, Faten A. Mostafa

**Affiliations:** 1 Chemistry of Natural and Microbial Products Department, National Research Centre, Dokki, Cairo Egypt; 2 Molecular Biology Department, National Research Centre, Dokki, Cairo Egypt

**Keywords:** Statistical factorial designs, β-galactosidase, Agro-industrial wastes, Purification, Thermodynamics

## Abstract

**Background:**

The main obstacle facing the utilization of microbial enzymes in industrial applications is the high cost of production substrates. As a result of the mentioned different wastes (coffee powder waste, dates nawah powder, molokhia stems, pea peels, lemon peels, and corn cobs) were investigated as low-cost nutritional substrates for the production of microbial β-galactosidase in this study. The purification of the enzyme and its kinetic and thermodynamics were investigated.

**Results:**

β-galactosidase was effectively produced by *Aspergillus foetidus* utilizing lemon peels and coffee powder waste by solid-state fermentation technique. The production yield was improved through Plackett-Burman Design declaring the significant effect of lemon peels and coffee waste powder, and beef extract quantities on *A. foetidus* β-galactosidase production. Followed by Central Composite Design investigating each factor with five levels resulting in 37363.1 U.ml^− 1^ production. The enzyme was fully purified by gel filtration technique through Sephadex G-150 giving one band with a molecular weight 40 KDa on SDS-PAGE gel. The maximal β-galactosidase activity was obtained at 50 °C with 0.4% ONPG. Cu^2+^, Fe^2+^, and Hg^2+^ showed severe inhibitory effect on pure enzyme activity. Energy required for enzyme activation (E_a_) and denaturation (E_d_) were determined to be 17.40, and 43.86 KJ.mol^− 1^, respectively. Parameters reflecting β-galactosidase thermal stability at 40, 45, and 50 °C as T_1/2_ and D-values values were determined to be 283.92, 209.43, and 168.56 min, and 943.34, 695.84, and 560.06 min, respectively.

## Introduction

β-Galactosidase (EC 3.2.1.23), β-gal, lactase, is an enzyme responsible for the hydrolysis of O-glycosidic bonds of lactose into glucose and galactose [1]. Offering a magic solution for lactose intolerance a nutritional disorder with 70% of the world’s population [2]. Yeasts, fungi, and bacteria all have β-galactosidase. Almonds, peaches, apples, and apricots are the primary plant sources of it [3]. Microbial β-galactosidases have drawn extensive attraction due to their high yields, high activity, and abundance [4]. *Aspergillus* species are common β-galactosidase producers that are generally recognized as safe’’ (GRAS) by FDA [5].

*β*-galactosidase is produced intracellularly by most of bacteria and yeast, while it is produced extracellularly by fungi. Intracellular *β*-galactosidases have high thermal and pH -sensitivity, as well as the extracellular form is acid and thermolabile [6].

In food industries, *β*-galactosidase improves the quality of ice creams and condensed milk by the reduction of crystallization due to high lactose concentration beside the production of lactose-free products. β-galactosidase is involved in the production of industrially important products such as ethanol and biosensors [3]. β-galactosidase plays an important role in health care through the synthesis of galacto-oligosaccharides (GOS) through transglycosylation. GOSs are nondigestible prebiotics that support the growth of *Lactobacillus* and *Bifidobacterium* species (beneficial bacteria) in the intestine that are very important for human health [3, 5].

Several studies showed the purification of fungal β-galactosidases via techniques like chromatography on DEAE-cellulose, ammonium sulfate fractionation, DEAE-Sephadex column chromatography [3].

One of the most critical obstacles that face the industrial application of microbial enzymes is the high cost. Recently to overcome this obstacle there has been a big attend to utilize the wastes accumulated from agricultural and industrial fields. Several enzymes of industrial potency have been produced utilizing agro-industrial wastes [7, 8]. Abdel Wahab et al. [9] utilized rice straw and orange peel wastes as cheap and eco-friendly substrates.

In this study, the main object was to lower the production cost of β-galactosidase via utilizing different low-cost wastes for the production by isolated micro-organisms (bacteria and fungi). Improving the β-galactosidase yield via the optimization of the most effective factors on β-galactosidase production via Plackett– Burman Design (PBD) followed by Central Composite Design (CCD) was carried out. At the last step, produced β-galactosidase was purified and studied for its physiochemical, kinetic and thermodynamic characteristics.

### Isolation of microorganisms

Rotten yogurt and milk whey were used as sources for the isolates. Loopful from each was streaked with a needle on nutrient agar (NA) and potato dextrose agar (PDA) plates and incubated at 28–30 °C. To purify the isolates, the colony (bacteria) and mycelium tips (fungi) are repeatedly transferred into NA (bacteria) and PDA (fungi) slants until the colony was deemed uniform. The pure isolates are maintained at 4 °C on NA (bacteria) and PDA (fungi) slants.

### β- galactosidase (β-gal) production screening

Agro-industrial wastes including coffee powder waste (CPW), dates nawah powder (DNP), molokhia stems (MS), pea peels (PP), lemon peels (LP), and corn cobs (CC) were used as carbon source for β-galactosidase production by the microbial isolates employing solid-state fermentation (SSF) and sub-merged fermentation (SMF) techniques. In the SSF 1 g of the waste substrate was moistened with 10 ml of distilled water (pH 5.0) in 250 ml Erlenmeyer flasks covered with hydrophobic cotton and autoclaved at 121 °C for 20 min. Flasks were inoculated with 1.0 ml inoculum containing (6 × 10^8^ spores·ml^− 1^) of 5 days old culture in the case of fungi and 24 h old culture in the case of bacteria which was prepared by harvesting the slant in 10 ml sterile distilled water. The inoculated flasks were incubated for 7 days at 30 °C under static conditions. At the end of the fermentation period, the enzyme was extracted by the addition of 50 ml of distilled water to each flask in a shaking incubator (30 min, 150 rpm).

In the SMF the waste was suspended in 50 ml of distilled water in 250 ml Erlenmeyer flasks undergoing the same conditions of sterilization and cultivation mentioned above. The inoculated flasks were incubated for 7 days at 30 °C in a shaking incubator 150 rpm. At the end of the fermentation period, the flasks were filtered through nylon cloth to take the fermented liquid broth followed by centrifugation as β-galactosidase source.

### Identification of the most promising isolate

β-galactosidase producing fungal isolate was identified through specific gene detection technique employing 18 S rRNA as molecular marker. At first genomic DNA extraction was performed through high performance fungal DNA, E.Z.N.A.^®^HP Fungal DNA Kit (D3195-01 50 preps). Followed by the preparation of the PCR master mixture in which small ribosomal subunit (18 S) rRNA primers (5’-CCTGGTTGATCCTGCCAGTA-3’) (5’-GCTTGATCCTTCTGCAGGTT-3’) Melchers et al. [10] was added. The total genomic DNA was amplified through GeneAmp Polymerase Chain Reaction (PCR) system cycler. The amplification products were detected through agarose gel electrophoresis and resultant PCR products was purified with Micro spin filters and quantities spectrophotometrically. Sequence analysis was employed using the ABI PRISM^®^ 3100 Genetic Analyzer (Micron-Corp.Korea). Gel documentation system (Geldoc-it, UVP, England), was applied for data analysis using Totallab analysis software, ww.totallab.com, (Ver.1.0.1). Aligned sequences were analyzed on NCBI website (http://www.ncbi.nlm.nih.gov/webcite) using BLAST to confirm their identity. The Genetic distances and MultiAlignments were computed by Pairwise Distance method using ClusteralW software analysis (www.ClusteralW.com). The fungal isolate was identified as *Aspergillus foetidus* isolate Gfwss and deposited at Genbank with accession number OR668926.

### β-galactosidase activity assay

The assay was carried out according to Abdel-wahab et al. [9] method, where 0.5 ml of both enzyme and substrate (15 mM o-nitrophenyl β-D-gal- actopyranoside, ONPG in 0.1 M sodium phosphate buffer, pH 7.0) were incubated at 37 ^◦^C for 20 min. 2 ml of 0.1 M sodium carbonate was used to stop the reaction. Absorbance was measured at wavelength 420 nm with a spectrophotometer. One unit of β-galactosidase expresses the amount of enzyme that produces one µmol of o-nitrophenol / min following the standard assay conditions.

### Plackett-Burman design (PBD)

In this step the effect of 19 factors namely, A: LP, B: DNP, C: CW, D: glucose, E: lactose, F: galactose, G: MgSO_4_, H: CuSO_4_, J: CaCl_2_, L: whey, M: bakers yeast, N: (NH_4_)_2_SO_4_, O: peptone, P: NaNO_3_, Q: KH_2_PO_4_, R: ZnSO_4_, S: KCl, T: beef extract on β-galactosidase production was assayed. Each factor was studied with low (-1) and high (+ 1) level producing 20 runs. The success of the design was analyzed with ANOVA.

### Central composite design (CCD)

The quantitive effect of the most effective factors A: LP (g.flask^− 1^); B: CW (g.flask^− 1^) and C: beef extract (g.l^− 1^) on ß-galactosidase production was analyzed each with five levels − 1.682, -1.00, 0, 1.00, 1.682 giving 20 runs. The success of the design was analyzed with ANOVA.

### Purification of *A. foetidus* β-galactosidase

Purification was achieved through gel filtration chromatography: Concentrated β-galactosidase crude culture filtrate (activity 1233961U, protein 484.8 mg, and specific activity 2545.3 U.mg^− 1^ protein) by lyophilization (to reach 5 ml) was loaded on Sephadex G-150 column pre-equilibrated with sodium phosphate buffer (0.1 M, pH 7.0). Fractions (5 ml) containing β-galactosidase activity were pooled investigated for protein and β-galactosidase content.

### Determination of protein content

Protein content was determined colorimetrically at 595 nm using coomassie brilliant blue G-250 (CBB) and bovine serum albumin (BSA) as a standard protein for protein curve, according to Bradford [11] protein solution containing 10 to 100 µg protein in a volume up to 0.1 ml was pipetted into 12 × 100 mm test tubes. The volume in the test tube was adjusted to 0.1 ml with appropriate buffer. Five milliliters of protein reagent was added to the test tube and the contents mixed either by inversion. The absorbance at 595 nm was measured after 2 min and before 1 h in 3 ml cuvettes against a reagent blank prepared from 0.1 ml of the appropriate buffer and 5 ml of protein reagent.

### Electrophoresis conditions

Samples were loaded into sodium dodecyl sulfate-polyacrylamide gel (SDS-PAGE) (12%) along with molecular weight marker. After electrophoresis, the gel was subjected to fixing solution (50% methanol + 10% glacial acetic acid), stained with staining solution (0.1% Coomassie Brilliant Blue R-250 dissolved in 50% methanol, and 10% glacial acetic acid) for 1 h. The gel was finally destained with destaining solution (40% methanol + 10% glacial acetic acid) [12].

### Physiochemical characterization

Investigating the optimum conditions of, temperature for the maximum β-galactosidase activity was achieved by incubating the reaction mixture (enzyme and ONPG) at different temperatures (25, 30, 40, 50, 60, and 70 °C), and substrate (ONPG) concentration by incubating the enzyme with different ONPG concentrations (0.1, 0.2, 0.3, 0.4, 0.6, 0.8, 1.00, 1.2, and 1.4%. The positive and negative effect of different metal ions (Cu^2+^, Fe^2+^, Na^+^, Zn^2+^, Mg^2+^, Ni^+^, K^2+^, Mn^2+^, Hg^2+^, Ba^2+^, Co^+^, Ca^2+^) and SDS, and EDTA with concentration 1 mM on β-galactosidase activity was investigated by pre-incubation of the enzyme with the metal for 30 min prior the reaction and compared to the pure enzyme activity (100%). The pre-incubation of the enzyme investigated the thermal stability of the pure β-galactosidase at 40, 45, and 50 °C for different times 15, 30, 45, and 60 min.

### Kinetic and thermodynamic characterization

Lineweaver-Burk plot was employed to analyze K_m_, V_max_ values. Activation energy E_a_, and activation energy of denaturation E_d_ were determined via Arrhenius plot.

The following equations were used to determine the denaturation parameters for pure *A. foetidous* β-galactosidase:

T_1 */* 2_ (half-life) = ln 2/K_d_.

D- value (decimal reduction time) = ln 10 */* K_d_.

ΔH_d_ (enthalpy) = E_d_ - RT.

ΔG_d_ (Gibbs native energy) = - RT⋅ln(K_d_⋅h */* K_b_⋅T).

ΔS_d_ (entropy) = (ΔH_d_ - ΔG_d_)*/* T.

where T is the corresponding absolute temperature (K), R is the gas constant (8.314 J mol^− 1^ K^− 1^), h is the Planck constant (6.626 × 10^− 34^ J min), K_b_ is the Boltzman constant (1.38 × 10^− 23^ J K^− 1^) and K_d_ is the deactivation rate constant (min^− 1^) [9].

## Results and discussion

### Screening of β-galactosidase production

Table 1 declared the following, firstly: the ability of different microbial isolates (bacteria, fungi) to utilize different agro-industrial wastes (LP, PP, CWP, DNP, CC, MS) for β-galactosidase production by both SSF and SMF with different degrees. Secondly: bacterial isolates produced β-galactosidase more effectively by SMF in contrast to fungal isolates that prefered SSF. Compared with SMF, SSF has many advantages, such as superior productivity, greater simplicity, lower capital investment, less energy requirement and wastewater output, and better product recovery, and is reported to be the most appropriate process for developing countries [13]. Thirdly: There are only two bacterial and two fungal isolates that can produce β-galactosidase utilizing LP, PP, DNP, and CWP. Martarello et al. [14] utilized soybean residue for the production of β-gal by *Aspergillus niger* isolated from Brazilian soils, Akcan [15] utilized rice bran for the production of β-galactosidase by *Bacillus licheniformis*, Bassetto et al. [16] utilized wheat bran for the production of β-galactosidase by *Penicillium* sp. Ali et al. [17] utilized rice flour, wheat flour and corn flour for β-galactosidase production by yogurt bacterial isolate with more efficiency on rice flour. Abdel Wahab et al. [9] utilized rice straw and orange peel for *Lactobacillus paracasei*.

**Table 1:** Screening for β-galactosidase production by different isolates utilizing different agro-industrial wastes by SMF and SSF techniques

Waste Isolate	CPW (U.ml^-1^)		DNP(U.ml^-1^)		MS (U.ml^-1^)		PP(U.ml^-1^)		LP(U.ml^-1^)		CC(U.ml^-1^)	
	SMF	SSF	SMF	SSF	SMF	SSF	SMF	SSF	SMF	SSF	SMF	SSF
B1	0	0	0	0	0	0	0	0	0	0	0	0
B2	0	0	0	0	0	0	0	0	0	0	0	0
B3	0	0	0	0	0	0	0	0	0	0	0	0
B4	0	0	0	0	0	0	0	0	0	0	0	0
B5	0	0	0	0	0	0	0	0	0	0	0	0
B6	0	0	0	0	0	0	0	0	0	0	0	0
B7	0	0	0	0	0	0	100.35	0	125.35	0	0	0
B8	0	0	150.25	0	0	0	0	0	130.55	0	0	0
F1	0	0	0	0	0	0	0	600.15	500.98	650.35	0	0
F2	0	800	0	690.33	0	0	0	0	0	720	0	0

### PBD for β-galactosidase production

**Fig. 1:**
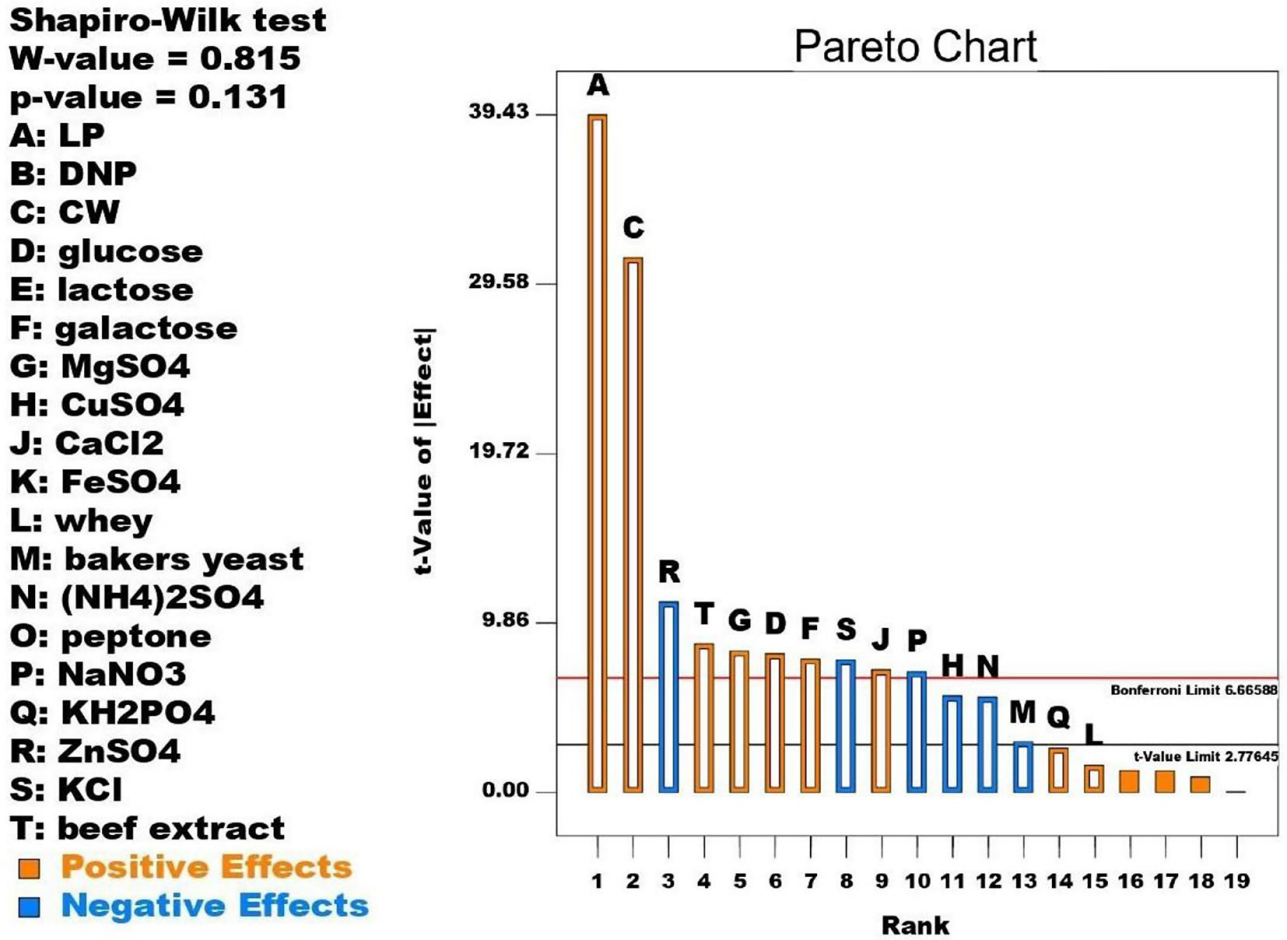
Pareto chart showing significant factors on β-galactosidase production by *A. foetidus*

Statistical optimization tools use randomly designed experimental runs due to carry out influence of full factorial design with less labor at shorter time [18, 19]. As shown in Table 2 the interaction between the tested 19 factors resulted in 20 runs and a wide variation in β-galactosidase production 0-13527.67 U.ml^− 1^. Of the tested factors 15 have a significant effect on β-galactosidase production and this effect was distinguished into positive (promotive, orange color) and negative (suppressive, blue color) effect as shown in Fig. 1 (Pareto chart). LP, CWP, beef extract, MgSO_4_, glucose, galactose, CaCl_2_, KH_2_PO_4_, and whey have positive stimulating effect while CuSO_4_, bakers yeast, (NH_4_)_2_SO_4_, NaNO_3_, ZnSO_4_, KCl have negative effect on β-galactosidase production. The β-galactosidase production can be calculated from the following equation:

β-galactosidase activity (U.ml^− 1^) = + 608.59 + 256.26 * LP + 202.08 * CWP + 52.49 * glucose + 50.51* galactose + 53.45* MgSO_4_ -36.52* CuSO_4_ + 46.35* CaCl_2_ + 10.32 * whey-19.15 * bakers yeast − 36.13 * (NH_4_)_2_SO_4_ -45.70 * NaNO_3_ + 16.80 * KH_2_PO_4_ -72.04* ZnSO_4_ -50.15 * KCl + 56.21 * beef extract.

**Table 2:** PBD for β-galactosidase production by *Aspergillus foetidus*

	Factor 1	Factor 2	Factor 3	Factor 4	Factor 5	Factor 6	Factor 7	Factor 8	Factor 9	Factor 10	Factor 11	Factor 12	Factor 13	Factor 14	Factor 15	Factor 16	Factor 17	Factor 18	Factor 19	β- galactosidaseactivity U.ml-^1^
Run	A: LP	B: DW	C: CWP	D: glucose	E: lactose	F: galactose	G: MgSO_4_	H: CuSO_4_	J: CaCl_2_	K: FeSO_4_	L: whey	M: bakers yeast	*N*: (NH_4_)_2_SO4	O: peptone	*P*: NaNO_3_	Q: KH_2_PO4	*R*: ZnSO_4_	S: KCl	T: beef extract	
	g.flask^− 1^	g.flask^− 1^	g.flask^− 1^	g.l^− 1^	g.l^− 1^	g.l^− 1^	g.l^− 1^	g.l^− 1^	g.l^− 1^	g.l^− 1^	ml.l^− 1^	g.l^− 1^	g.l^− 1^	g.l^− 1^	g.l^− 1^	g.l^− 1^	g.l^− 1^	g.l^− 1^	g.l^− 1^	
1	1	0	1	0	0	0	0	0.05	0.5	0	1	5	0	0	2	5	0.02	0.5	0	7184.04
2	1	0	0	5	5	5	0.5	0	0.5	0	1	1	0	0	0	5	0.02	0	2	10418.88
3	0	0	1	5	5	5	0	0.05	0	0.02	0	1	0	0	2	5	0	0.5	2	6092.282
4	0	0	0	5	5	0	0.5	0.05	0	0	1	5	2	2	0	5	0	0.5	0	951.5821
5	1	0	1	0	5	0	0	0	0	0.02	1	1	2	2	0	1	0.02	0.5	2	8626.24
6	1	1	0	5	5	0	0	0.05	0.5	0.02	1	1	2	0	2	1	0	0	0	6280.981
7	1	0	0	0	0	5	0.5	0	0.5	0.02	0	1	2	2	2	5	0	0.5	0	7130.1265
8	1	1	1	5	0	5	0	0.05	0	0	0	1	2	2	0	5	0.02	0	0	9967.35075
9	0	1	1	0	0	5	0.5	0.05	0.5	0	1	1	2	0	0	1	0	0.5	2	7103.1695
10	0	0	1	5	0	5	0.5	0	0	0.02	1	5	2	0	2	1	0.02	0	0	5101.61225
11	1	1	1	0	5	0	0.5	0	0	0	0	5	2	0	2	5	0	0	2	10850.1925
12	0	0	0	0	5	5	0	0.05	0.5	0	0	5	2	2	2	1	0.02	0	2	0
13	0	1	1	0	5	5	0	0	0.5	0.02	1	5	0	2	0	5	0	0	0	7595.13475
14	0	1	1	5	5	0	0.5	0	0.5	0	0	1	0	2	2	1	0.02	0.5	0	5020.74125
15	1	1	0	0	5	5	0.5	0.05	0	0.02	0	5	0	0	0	1	0.02	0.5	0	4987.045
16	1	0	1	5	0	0	0.5	0.05	0.5	0.02	0	5	0	2	0	1	0	0	2	13525.67475
17	0	0	0	0	0	0	0	0	0	0	0	1	0	0	0	1	0	0	0	1018.9746
18	1	1	0	5	0	5	0	0	0	0	1	5	0	2	2	1	0	0.5	2	7514.26375
19	0	1	0	0	0	0	0.5	0.05	0	0.02	1	1	0	2	2	5	0.02	0	2	1114.67195
20	0	1	0	5	0	0	0	0	0.5	0.02	0	5	2	0	0	5	0.02	0.5	2	1234.6306

The success of the design was statistically analyzed and confirmed via ANOVA as shown in Table 3. The Model F-value of 210.22 implied that the model was significant. Values of “Prob > F” less than 0.05 indicate model terms are significant. In this case A, C, D, F, G, H, J, M, N, P, R, S, T were significant model terms. R^2^ value 0.9987 implies that the design can explain 99.87% of the results. The Predicated R^2^ of 0.9683 is in reasonable agreement with the Adjective R^2^ of 0.9940.

**Table 3:** Statistical analysis (ANOVA) for PBD for β-galactosidase production

Source	Sum of squares	df	Mean square	F value	*p*-value	
					Prob > F	
Model	266323795.8	15	17754919.72	210.2203	< 0.0001	significant
A-LP	131338356	1	131338356	1555.061	< 0.0001	
C-CWP	81669751.97	1	81669751.97	966.9793	< 0.0001	
D-glucose	5510823.96	1	5510823.96	65.24879	0.0013	
F-galactose	5102657.336	1	5102657.336	60.41605	0.0015	
G-MgSO_4_	5713589.438	1	5713589.438	67.64956	0.0012	
H-CuSO_4_	2667420.179	1	2667420.179	31.58256	0.0049	
J-CaCl_2_	4295870.48	1	4295870.48	50.8636	0.0020	
L-whey	212913.6532	1	212913.6532	2.520922	0.1875	
M-bakers yeast	733154.6573	1	733154.6573	8.680636	0.0421	
N-(NH_4_)_2_SO_4_	2610626.516	1	2610626.516	30.91012	0.0051	
P-NaNO_3_	4176770.56	1	4176770.56	49.45344	0.0022	
Q-KH_2_PO_4_	564543.8586	1	564543.8586	6.684264	0.0610	
R-ZnSO_4_	10378325.43	1	10378325.43	122.8806	0.0004	
S-KCl	5029394.927	1	5029394.927	59.54862	0.0015	
T-beef extract	6319596.831	1	6319596.831	74.82475	0.0010	
Residual	337834.5518	4	84458.63795			
Cor Total	266661630.3	19				

Rashmi and Siddalingamurthy [20] tested the interaction between 11 factors (Tamarind seed powder TSP, pH, NaNO_3_, (NH_4_)_2_SO_4_, yeast extract, urea, lactose, maltose, MgSO_4_, KH_2_PO_4_, and cellobiose exploring that NH_4_(SO_4_)_2_, lactose, and MgSO_4_ had a significant positive influence and pH, yeast extract, maltose, and NaNO_3_ had significant negative influence. Akcan [15] found that the supplementation of the production medium with metabolizable sugars (mannose, xylose, lactose, sucrose, fructose, galactose, glucose, and arabinose) suppressed the β-galactosidase production by *Bacillus licheniformis* in accordance with Konsoula and Kyriakides [21]. The supplementation of the medium with nitrogen sources (organic and inorganic) showed variable effects. i.e. Beef extract exerted a positive significant effect on β-galactosidase production by the fungal isolate in contrast with Chandel and Sharma [22] while bakers yeast exerted negative significant effect. Akcan [15] found that the supplementation of the production medium with different organic (peptone, tryptone, yeast extract, beef extract, urea, and casein) and inorganic (ammonium nitrate, sodium nitrate, ammonium chloride and ammonium sulfate) nitrogen sources adversely influenced the production of β-galactosidase.

Chandel and Sharma [22] found that β-galactosidase is an inducible enzyme generally induced in lactose. Other carbon sources such as glucose, maltose and sucrose failed to significantly induce β-galactosidase production. Yeast extract, (NH_4_)_2_SO_4_ and KH_2_PO_4_ were reported as significant factors for β-galactosidase production by *Kluyveromyces* sp. CK8 [23]. Deng et al. [24] used PBD to test the effect of pH, temperature, and lactose on *Lactobacillus leichmannii* β-galactosidase production. da Silva et al. [25] used a complete factorial design to evaluate the interaction of the fermentation time, temperature, pH, and lactose concentration on the production of *Enterococcus faecium* β-galactosidase. β-galactosidase production by *Bacillus megaterium* NM56 was enhanced when galactose or lactose was used as a carbon source while sucrose or glucose significantly inhibited β-galactosidase synthesis when used as a carbon source [26]. The enhancement effect of tryptone and yeast extract followed by peptone and beef extract was also reported on *Bacillus megaterium* β-galactosidase production [26]. Ali et al. [17] found that the addition of yeast extract, tryptone and peptone repressed β-galactosidase production. According to Afolabi et al. [27] glucose decreased β-galactosidase production by *Kluyveromyces marxianus* while yeast extract and urea were considered appropriate nitrogen sources. Abdel Wahab et al. [9] studied the interaction effect between wheat bran, rice straw, orange peel, whey, peptone, yeast extract, lactose, glucose, KH_2_PO_4_, MgSO_4_, and CaCl_2_ on *L. paracasei* β-galactosidase production through PBD and found that orange peel, rice straw, and KH_2_PO_4_ were the most influencing factors causing a positive effect on enzyme production followed by lactose, wheat bran, and MgSO_4_.

### CCD for β-galactosidase production

**Fig. 2:**
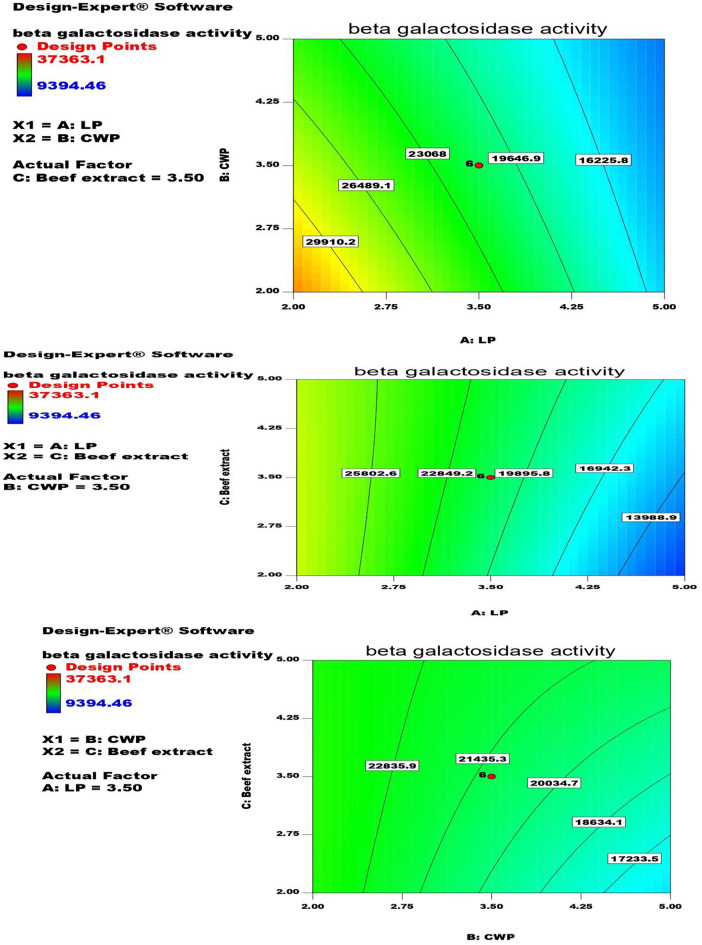
The interaction between the most effective factors A: LP (g.flask^− 1^); B: CWP (g.flask^− 1^) and C: beef extract (g.l^− 1^) on β-galactosidase production

The interaction between the most effective factors A: LP (g.flask^-1^); B: CWP (g.flask^-1^) and C: beef extract (g.l^-1^) on ß-galactosidase production as shown in Fig. 2; Table 4 led to wide variation 9394.46- 37363.1 U.ml^-1^. The highest ß-galactosidase production was achieved in run 15 with 2 g.flask^-1^ of each LP and CW with 2 g.l^-1^ of beef extract causing 2.76-fold increase compared with PB optimization. β-galactosidase production can be calculated from the following equation:

β-galactosidase activity (U.ml-^1^) = + 21165.72 -7434.64 * LP -2828.61* CWP + 1246.17 * beef extract + 1540.46 * LP * CWP + 1393.78 * LP * beef extract + 1312.86 * CWP * beef extract + 125.69 * LP 2 + 236.14 * CWP 2 -181.36 * beef extract 2.

**Table 4:** CCD for β-galactosidase production by *A. foetidus*

Run	Factor 1 A: LP g.flask^− 1^	Factor 2 B: CWP g.flask^− 1^	Factor 3 C: Beef extract g.l^− 1^	β-galactosidase activity U.ml^− 1^
1	3.5	0.977311	3.5	25053.6
2	3.5	3.5	3.5	21205.11
3	5	5	5	14797.61
4	2	5	2	23036.61
5	0.977311	3.5	3.5	31771.55
6	3.5	6.022689	3.5	17236.02
7	3.5	3.5	0.977311	17567
8	2	2	5	31939.67
9	3.5	3.5	3.5	21205.11
10	3.5	3.5	3.5	21205.11
11	3.5	3.5	3.5	21205.11
12	2	5	5	27363.1
13	3.5	3.5	3.5	21205.11
14	3.5	3.5	3.5	21205.11
15	2	2	2	37363.1
16	3.5	3.5	6.022689	22360.9
17	6.022689	3.5	3.5	9893.248
18	5	2	2	13060.64
19	5	2	5	17710.8
20	5	5	2	9394.46

The design was statistically analyzed via ANOVA (Table 5). The success of the design was emphasized with R^2^, adjective R^2^, and predicated R^2^ values (0.9730, 0.9486, and 0.7783, respectively) which employed that 97.30% of the results can be explained by the design. The Model F-value 39.98 implied the significance of the design. Prob > F values less than 0.0500 indicated that the selected factors were significant including A, B, C, AB, AC, BC.

**Table 5:** Statistical analysis (ANOVA) for CCD

Source	Sum of Squares	df	Mean Square	F Value	*p*-value Prob > F	
Model	9.34E + 08	6	1.56E + 08	73.23514	< 0.0001	significant
A-LP	7.55E + 08	1	7.55E + 08	355.2655	< 0.0001	
B-CWP	1.09E + 08	1	1.09E + 08	51.42583	< 0.0001	
C-Beef extract	21208152	1	21208152	9.98127	0.0075	
AB	18984162	1	18984162	8.934585	0.0105	
AC	15541014	1	15541014	7.314124	0.0180	
BC	13788886	1	13788886	6.489514	0.0243	
Residual	27622334	13	2124795			
Lack of Fit	27622334	8	3452792			
Pure Error	0	5	0			
Cor Total	9.61E + 08	19				

Rashmi and Siddalingamurthy [20] used CCD to optimize the best level of lactose, (NH_4_)_2_SO_4_, and MgSO_4_ for *Aspergillus terreus* β-galactosidase production causing 2.8 -fold increase in comparison with basal medium. da Silva et al. [25] used a RSM to optimize the level of lactose, pH, and temperature for *Enterococcus faecium* β-gal production. Abdel Wahab et al. [9] optimized the orange peel, rice straw, and KH_2_PO_4_ level for *L. paracasei* β-galactosidase production. Martarello et al. [14] optimized the level of pH, agitation (rpm), and temperature for *Aspergillus niger* β-gal production. Deng et al. [24] optimized the level of pH, and lactose through CCD for *Lactobacillus leichmannii* β-gal production. Al- jazairi et al. [28] used Response Surface Methodology (RSM) as a statistical analysis to determine the initial sugar concentration, agitation speed, initial pH, incubation time and temperature for the optimization of β-galactosidase production in synthetic medium containing lactose as a carbon source for *Kluyveromyces marxianus* β-galactosidase production. Four culture medium parameters pH, lactose, casein, and inactive beer yeast were optimized through CCD to optimize *Lactobacillus reuteri* β-galactosidase production [29]. Am-aiam and Khanongnuch [23] optimized the level of yeast extract, KH_2_PO_4_, and (NH_4_)_2_SO_4_ through CCD for *Kluyveromyces* sp. β-galactosidase production.

### Purification of *A. foetidous* β-galactosidase

**Fig. 3:**
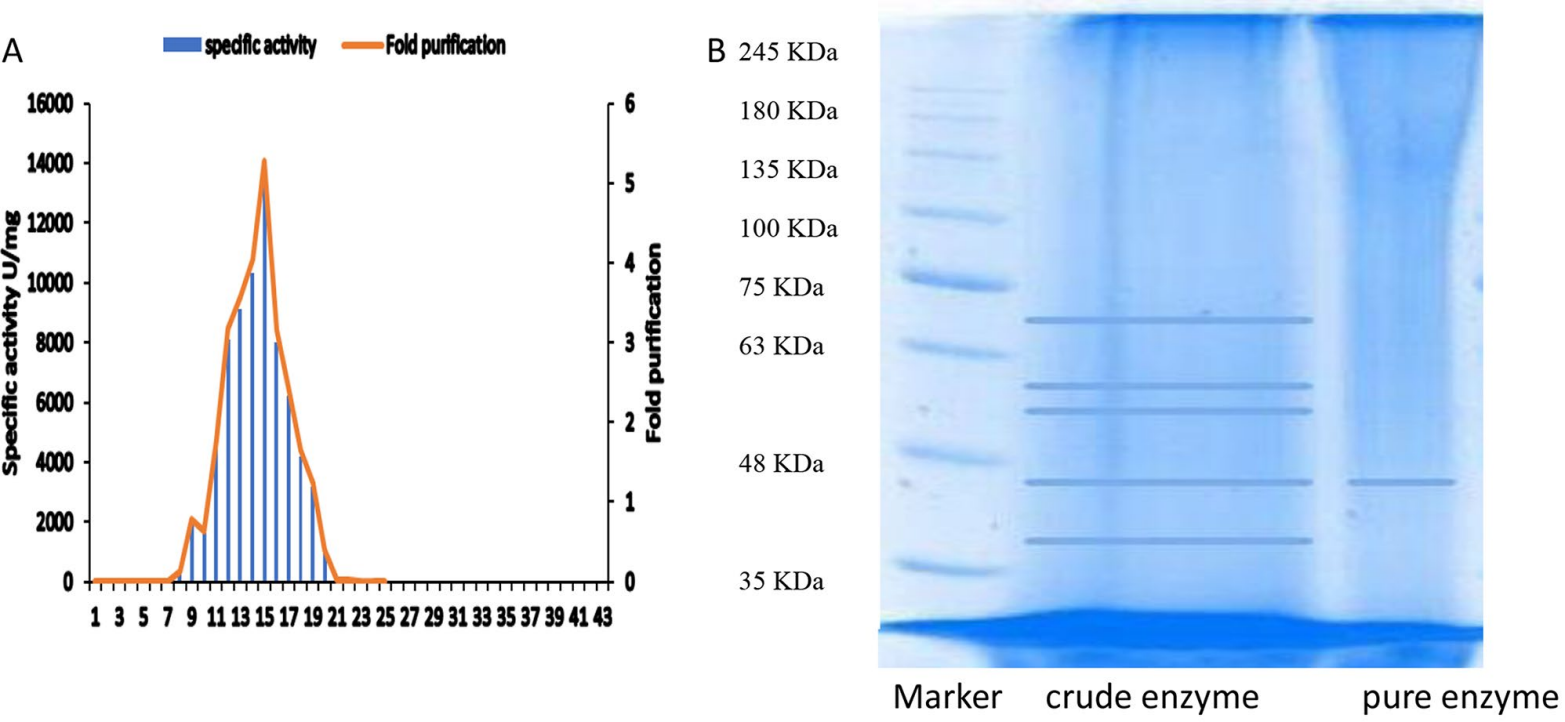
**A**, the purification profile for *A. foetidus* β-galactosidase through Sephadex G-150; **B**, SDS-PAGE gel showing the purity and molecular weight of *A. foetidus* β-galactosidase

As declared in Fig. 3A the elution of Sephadex G-150 with 0.1 M phosphate buffer pH 7.0 loaded with 1233961U of *A. foetidus* β-galactosidase succeeded in the purification of β-galactosidase in fractions 11–19 with the peak at 15 causing 5.27-fold purification (specific activity 13425.13063 U.mg protein^− 1^ compared with 2545.3 U.mg protein^− 1^ for crude β-galactosidase). The purity of the enzyme was emphasized with SDS gel (Fig. 3B) with a molecular weight 40 KDa. β-galactosidase showed a wide range of molecular weight from different sources. β-galactosidase from *Lactobacillus plantarum* HF571129 (heterodimer with a molecular weight of 60 kDa (larger subunit) and 42 kDa (smaller subunit)) [30] *Pediococcus pentosaceus* ID-7 (LacL (72.2 kDa) and LacM (35.4 kDa)) [31] *Aspergillus niger* (76 KDa) [14], *Streptococcus thermophilus* (116 KDa) [32].

### Physiochemical characterization

**Fig. 4:**
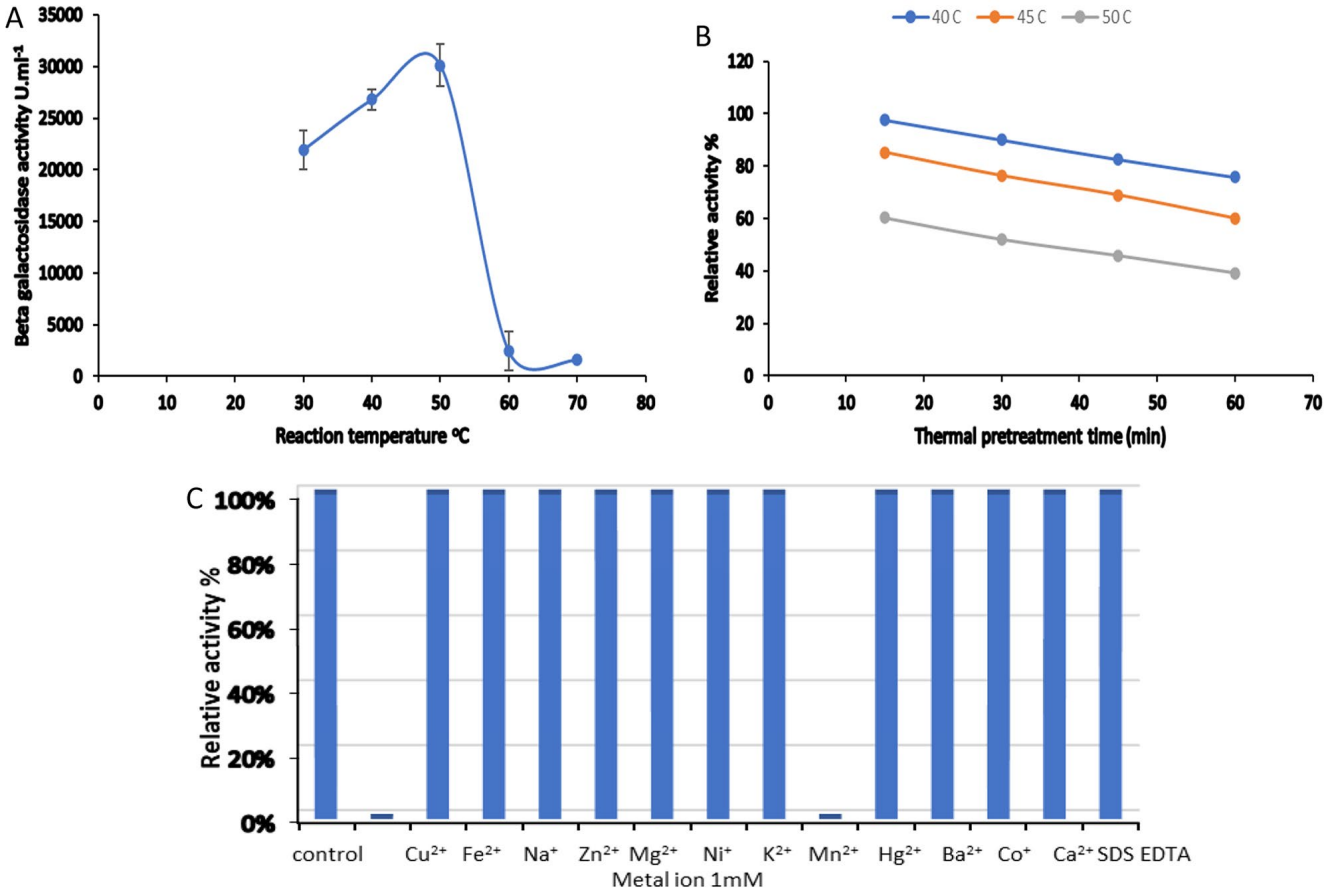
Physiochemical characterization of *A. foetidus* β-galactosidase, **A**: effect of reaction temperature; **B**: thermal pretreatment at different temperatures; **C**: effect of different metal ion

The optimum temperature for the maximum β -galactosidase activity (30124.56 U.ml^− 1^) as shown in Fig. 4A was found to be 50 °C nearly similar to β -galactosidases from *Pediococcus pentosaceus* ID-7 (50 °C) [31], *Aspergillus niger* (50 °C) [14], *B. paralicheniformis* 5NK (55 °C) [33], *Lactobacillus acidophilus* (45 °C) [34], higher than that for those from *Alteromonas* sp. ML52 (35 °C) [35], *Bacillus* sp. BY02 (40 °C) [36], *Steptococcus thermophillus* and *Escherichia coli* (40 and 30 °C, respectively) [32]. The thermal pretreatment of β-galactosidase (Fig. 4B) affected negatively on enzyme activity as the temperature and the duration of pretreatment was raised may be due to protein denaturation and destruction of enzyme active site. *(A) foetidous* β-galactosidase lost 24.3, 39.85, and 60.85% of its activity after pretreatment at 40, 45, and 50 °C, respectively for 60 min. According to Sun et al. [35] *Alteromonas* sp. ML52 β-galactosidase lost most of its activity after 30 min of incubation at 50 °C while *(B) subtilis* β- galactosidase retained 87% of its activity at 50 °C after 120 min of pretreatment [33].

The effect of metal ions on enzyme activity differs according to the type of metal ion and the origin of the enzyme as declared in Fig. 4C the pure β -galactosidase was not affected positively by any of the tested metals. In contrast, some of them especially Cu^2+^, and Fe^2+^ caused complete inhibition for the activity similar to that reported for β-galactosidase from *Alteromonas sp.* ML52 [35]. According to Liu et al. [37] both metal ions enhanced the catalytic activity of β-galactosidase from a thermophilic anaerobic bacterial consortium YTY-70. Martarello et al. [14]

revealed the stability of *A. niger* β-galactosidase in the presence of Zn^2+^, Ni^2+^, and Mg^2+^ ions. β-galactosidase from *Pediococcus pentosaceus* ID-7 [31] was strongly activated by Mg^2+^, Mn^2+^, and Zn^2+^. β-galactosidase from *Lactobacillus acidophilus* was inactivated in the presence of Ca^2+^, Ba^2+^, and Cu^2+^ [34]. Zhou et al. [36] reported significant effect for Zn^2+^, Mn^2+^, Mg^2+^, and Co^2+^ on *Bacillus* sp. BY02 β-galactosidase.

### Kinetics and thermodynamics characterization

**Fig. 5:**
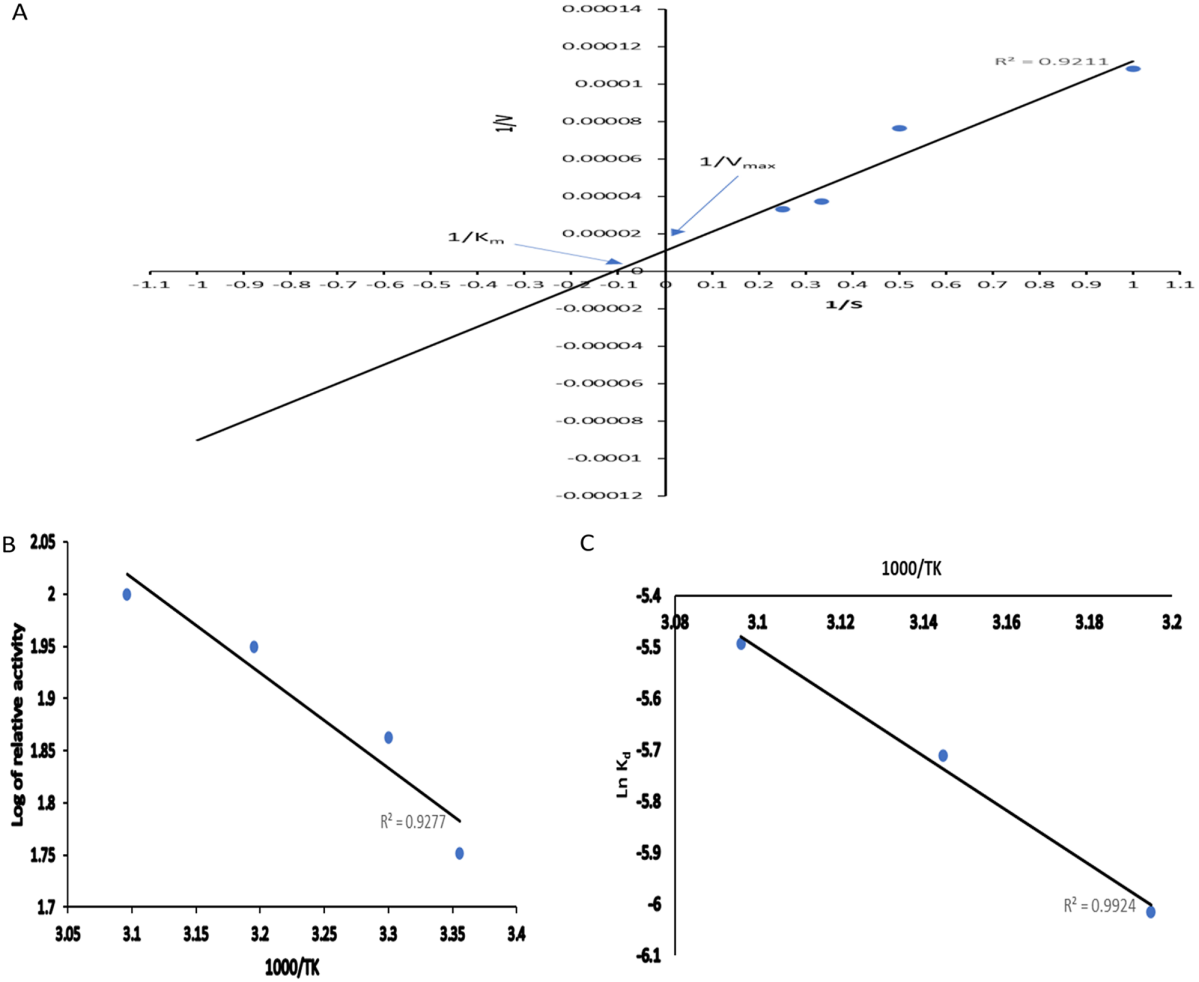
**A**, Lineweaver-Burk plot showing K_m_ and V_max_ values for *A*. *foetidus* β-galactosidase; **B** and **C**, Arrhenius plot showing the E_a_, E_d_ values for *A*. *foetidus* β-galactosidase

K_m_ is the substrate concentration at which the enzyme has the half maximum velocity. Referring to the Lineweaver-Burk plot (Fig. 5A) K_m_ and V_max_ were determined to be 10 mg.ml^− 1^ (equivalent to 33.20 mM) and 100,000 µmol.ml^− 1^.min^− 1^, respectively toward ONPG. K_m_ and V_max_ values are verified according to the substrate. β-galactosidase from *L. paracasei* showed K_m_ and V_max_ values 3.33 mM and 12236.61 mmol min^− 1^ mg^− 1^, respectively [38]. Selvarajan and Mohanasrinivasan [30] stated that *L. plantarum* β-galactosidase K_m_ and V_max_ values for ONPG were 6.644mM and 147.5 µmol min^− 1^ mg^− 1^, respectively while their counterparties toward lactose were 23.28 mM and 10.88 µmol min^− 1^ mg^− 1^, respectively indicating higher affinity toward ONPG.

Arrhenius plot (Fig. 5B and C) showed the E_a_, E_d_ to be 17.40, and 43.86 KJ.mol^-1^, respectively. E_a_ and E_d_ for any enzyme differ according to the source of the enzyme. β-galactosidase has E_a_ and E_d_ values from, *Lactobacillus paracasei* 16.92 and 157.38 kJ mol^-1^ [38], *Aspergillus oryzae* 18.53 kJ mol^−1^ and 278.00 kJ mol^−1^ [37]. K_d_, T_1/2_, D-values, ΔH_d_, ΔG_d_, and ΔS_d_ (Table 6) are thermodynamic parameters that reflect the thermal stability of the enzyme. These parameters differ according to the nature of the enzyme (crude, free (partial pure), immobilized or pure) and temperature. Abdel Wahab et al. [38] pointed that T_1/2_ and D- values for crude *L. paracasei* at 55, 60, 65 and 70 °C were higher than those for partially pure enzyme under the same conditions of heat pretreatment and these values decreases as the temperature and duration of heat pretreatment were prolonged due to protein denaturation. Marwa [39] stated that β‑galactosidase covalently bonded onto calcium pectinate‑agar beads had T_1/2_ and D- values at 60, 63, 65, and 70 °C higher than their counterparties for the free enzyme.

**Table 6:** Thermodynamics for the denaturation of *A. foetidus* β-galactosidase

Parameter Temperature °C	K_d_ (min^− 1^)	T_1/2_ (min)	D-value (min)	ΔH_d_(KJ.mol^− 1^)	ΔG_d_(KJ.mol^− 1^)	ΔS_d_ (J.mol^− 1^.k^− 1^)
**40**	0.002441	283.92	943.34	41.26	92.44	-163.51
**45**	0.00331	209.43	695.84	41.22	93.15	-163.31
**50**	0.004112	168.56	560.05	41.17	94.07	-163.77

## Conclusion

*Aspergillus foetidus* isolate Gfwss utilized effectively lemon peels and coffee waste powder as nutritional substrates for the production of β-galactosidase. The enzyme production was maximized 46.70 times via 2 steps statistical designs. The maximum *A. foetidus* β-galactosidase productivity was obtained in a medium composing of LP, 2 g.flask^− 1^; CWP, 2 g.flask^− 1^,glucose 5 g.l^− 1^, MgSO_4_, 0.5 g.l^− 1^, CuSO_4_, 0.05 g.l^− 1^; CaCl_2_, 0.5 g.l^− 1^; FeSO_4_, 0.02 g.l^− 1^; bakers yeast, 5 g.l^− 1^; peptone, 2 g.l^− 1^; KH_2_PO_4_, 1 g.l^− 1^, beef extract, 2 g.l^− 1^ by SSF after 7 days of incubation. The elution through Sephadex G-150 gel column fulfilled in the purification of the enzyme with 5.27-fold purification as evidenced by SDS-gel electrophoresis as a single band. The purified *A. foetidus* β-galactosidase was active from 30 to 60 °C. The enzyme activity was not enhanced by any of the tested metal ions Cu^2+^, Fe^2+^, Na^+^, Zn^2+^, Mg^2+^, Ni^+^, K^2+^, Mn^2+^, Hg^2+^, Ba^2+^, Co^+^, Ca^2+^ and SDS, and EDTA. On country, they have adverse effect on it with varying degree. The thermostability of pure *A. foetidus* β-galactosidase decreased as the temperature was elevated as the values of D-values, T_1/2_, ΔH_d_ and ΔG_d_ at 45 were 209.43 min, 695.84 min, 41.22 KJ.mol^− 1^ and 93.15 KJ.mol^− 1^, respectively higher their counterparties at 50 °C (168.56 min, 560.06 min, 41.74 KJ.mol^− 1^, and 94.07 KJ.mol^− 1^, respectively). *A. foetidous* β-galactosidase can be utilized in food industry for the preparation of GOS as prebiotics and anti-oxidants ingredients and this is our target in the coming part of our project.

## Data Availability

No datasets were generated or analysed during the current study.
